# Status, challenges, and future prospects of veterinary vaccines for sustainable livestock production in Bangladesh

**DOI:** 10.14202/vetworld.2026.1300-1321

**Published:** 2026-03-23

**Authors:** Md. Zahangir Hosain, Tahmina Begum, Md. Bayzer Rahman, Sharmin Sultana, Md. Mostofa Kamal

**Affiliations:** 1 Livestock Research Institute, Mohakhali, Dhaka-1212, Bangladesh; 2 Department of Livestock Services, Krishi Khamar Sarak, Farmgate, Dhaka-1215, Bangladesh

**Keywords:** Bangladesh, disease control, import dependency, livestock production, One Health, vaccine production, veterinary vaccines, zoonotic diseases

## Abstract

Veterinary vaccines are crucial tools for preventing infectious diseases, boosting animal productivity, and supporting sustainable livestock farming. This review examines the current status, challenges, and future outlook of veterinary vaccine development, production, and use in Bangladesh. The livestock sector in Bangladesh is vital for food security, rural livelihoods, and the national economy, contributing about 1.8% to the gross domestic product and supporting millions of farmers. Yet, the sector continues to suffer significant losses from infectious diseases such as foot-and-Mouth Disease, Peste des Petits ruminants, anthrax, hemorrhagic septicemia, Newcastle disease, and avian influenza. Therefore, effective vaccination programs are essential for disease control and improving livestock productivity. Bangladesh has gradually increased its veterinary vaccine production capacity through institutions such as the Livestock Research Institute and the Bangladesh Livestock Research Institute, with growing participation from private pharmaceutical companies. Over the past decade, vaccine production has risen from approximately 236 million doses in 2015–2016 to about 327 million doses in 2024–2025. Despite this progress, domestic production still falls short of meeting national demand. Current estimates show that locally produced vaccines cover only about 23%–27% of the demand for ruminant vaccines and roughly 6%–13% for poultry vaccines, leading to a heavy dependence on imported vaccines. Major challenges facing the veterinary vaccine sector include limited production capacity, outdated manufacturing infrastructure, lack of advanced vaccine technologies, regulatory hurdles, weak cold-chain logistics, and insufficient investment in research and development. Nevertheless, Bangladesh has significant opportunities to strengthen its veterinary vaccine ecosystem. Advances in molecular biology, recombinant vaccine technologies, genomic surveillance, and thermostable vaccine development offer promising pathways to improve vaccine efficacy and accessibility. Additionally, better collaboration between public institutions, academia, and private industry, along with supportive government policies and stronger regulatory frameworks, could greatly expand local vaccine production and lessen reliance on imports. Overall, strengthening veterinary vaccine research, manufacturing capacity, quality assurance systems, and distribution infrastructure is crucial for achieving sustainable livestock production in Bangladesh. Improved vaccine access and coverage will not only boost livestock productivity but also support food security, reduce economic losses, and contribute to national and global One Health goals.

## INTRODUCTION

Veterinary vaccines are essential for protecting animal health, increasing livestock productivity, minimizing economic losses, and preventing zoonotic diseases from spreading to humans [1]. Worldwide, vaccination is regarded as one of the most effective methods for controlling infectious animal diseases, including foot-and-mouth disease (FMD), Newcastle disease (ND), avian influenza (AI), rabies, peste des Petits Ruminants (PPR), lumpy skin disease (LSD), duck plague, and fowl cholera. The history of veterinary vaccines goes back to the late 18th century when Edward Jenner created the first smallpox vaccine in 1796, establishing the groundwork for immunization approaches in both human and veterinary medicine [2]. Later, Louis Pasteur’s groundbreaking research in the late 19th century led to the creation of the first animal vaccines, such as those for anthrax and rabies, which laid the scientific foundation for modern vaccine development [3].

The global veterinary vaccine market has grown quickly in recent decades due to increasing demand for animal-derived food products, heightened awareness of zoonotic diseases, and the need to ensure food safety under the One Health approach [4]. Modern veterinary vaccines include live attenuated, inactivated (killed), subunit, recombinant, vectored, and nucleic acid-based (DNA/RNA) vaccines. Advances in biotechnology, such as cell culture systems, recombinant DNA technology, and reverse genetics, have greatly enhanced vaccine safety, effectiveness, and production efficiency [2]. Currently, regions like North America, Europe, and the Asia–Pacific lead the global vaccine production industry. However, vaccine manufacturing capacity remains limited in many developing countries, which often rely on imported vaccines or donor-supported production systems. International organizations like the World Organization for Animal Health, Food and Agriculture Organization, and World Health Organization play vital roles in setting standards, ensuring quality, and improving global vaccine access. Additionally, the development of thermostable, low-cost, and easily administered vaccines is increasingly emphasized for low-resource areas. Overall, veterinary vaccine production is a crucial foundation for animal health, public health, and sustainable livestock production worldwide.

The livestock sector is vital for national food security, rural incomes, and economic growth in many developing countries, including Bangladesh, by providing essential animal-based foods like meat, milk, and eggs to a rapidly growing population [5, 6]. However, livestock production often faces challenges from infectious diseases such as FMD, PPR, anthrax, hemorrhagic septicemia (HS), ND, fowl cholera, and emerging diseases like LSD, which cause significant production losses, threaten farmers’ livelihoods, and pose risks to public health and international trade [7–9]. Therefore, reliable and affordable veterinary vaccines are crucial for disease prevention, boosting livestock productivity, and strengthening national preparedness within the One Health framework. Veterinary vaccination plays a key role in preventing zoonotic disease transmission, improving food safety, reducing antimicrobial resistance, enhancing public health surveillance, and supporting environmental sustainability. By controlling infectious agents at their source in animals, vaccination programs simultaneously protect human health, maintain ecological balance, and promote sustainable livestock economies [10].

In Bangladesh, veterinary vaccine production has traditionally been controlled by public institutions, especially the Livestock Research Institute (LRI) under the Department of Livestock Services (DLS), which makes vaccines for major livestock and poultry diseases such as PPR, FMD, LSD, HS, anthrax, ND, fowl cholera, and duck plague [11]. Despite these efforts, the country’s vaccine production capacity has not consistently met national demand, and locally made vaccines only cover a small part of the total need. As a result, Bangladesh depends heavily on imported vaccines, with an increasing role for the private pharmaceutical industry to fill supply gaps. The veterinary vaccine sector also faces several structural issues, including limited cold-chain infrastructure, shortages of raw materials, regulatory and quality control challenges, and a lack of skilled workers and distribution networks. These obstacles slow the growth of large-scale manufacturing facilities that follow Good Manufacturing Practices (GMP). Additionally, differences in farmers’ awareness, vaccine access, and vaccination habits weaken the overall success of immunization efforts, making it difficult for the country to become self-sufficient without coordinated investments and policy changes [6].

Despite the increasing importance of veterinary vaccination for livestock health and food security, comprehensive analyses of Bangladesh’s veterinary vaccine ecosystem remain scarce. Most studies focus on specific diseases, farmer knowledge and practices, or separate vaccination campaigns rather than examining the entire vaccine production and usage system holistically. The existing literature seldom connects the national disease burden with vaccine manufacturing capacity, regulatory systems, vaccination coverage, and import reliance. Additionally, systematic assessments comparing domestic vaccine production with national demand in the ruminant and poultry sectors are limited. Although institutions like LRI and DLS provide valuable production statistics, these data are rarely integrated with epidemiological evidence, vaccination coverage trends, and sectoral policies to offer a comprehensive view of the country’s vaccine landscape.

Additionally, limited attention has been paid to the technological readiness of vaccine development efforts in Bangladesh, including the shift from laboratory research to large-scale industrial production. Research on advanced vaccine platforms, such as recombinant, vectored, and nucleic acid-based vaccines, remains fragmented and is rarely assessed in relation to national manufacturing capabilities and regulatory infrastructure. There is also a lack of detailed evaluations of supply chain challenges, cold-chain management, quality assurance systems, and public–private collaboration in vaccine production and distribution. These gaps hinder policymakers and researchers from identifying priority areas to enhance domestic vaccine production and increase vaccination coverage. Therefore, a comprehensive review that combines production capacity, disease epidemiology, vaccine demand, import dependence, and future technological opportunities is essential to gain a clearer understanding of the veterinary vaccine sector’s development in Bangladesh.

This review aims to provide a thorough, evidence-based assessment of the current situation, challenges, and future outlook of veterinary vaccines in Bangladesh. Specifically, it consolidates available information on livestock disease burdens, national vaccination practices, vaccine production systems, and regulatory frameworks for veterinary biologics. It examines the roles of key institutions, including LRI, DLS, and the Bangladesh Livestock Research Institute, along with private sector manufacturers involved in vaccine development and distribution. The review also analyzes trends in vaccine production and vaccination coverage, compares domestic manufacturing capacity with national demand, and emphasizes the reliance on imported vaccines.

Furthermore, this study highlights key structural and technological barriers impacting vaccine research, development, manufacturing, quality assurance, and distribution in Bangladesh. Special emphasis is placed on opportunities to strengthen domestic production through improved infrastructure, advanced biotechnology platforms, enhanced regulatory oversight, and better collaboration among government agencies, academia, and industry. By combining epidemiological evidence with production and policy perspectives, this review aims to provide strategic insights that can guide national efforts to improve vaccine access, enhance disease prevention programs, and promote sustainable livestock production. Ultimately, the findings of this review are designed to support evidence-based policymaking and help develop a more resilient veterinary vaccine system aligned with national livestock development goals and the broader One Health framework.

## REVIEW METHODOLOGY

### Literature search strategy

A structured literature search was performed to gather scientific and institutional information related to veterinary vaccines and livestock health in Bangladesh. The search was carried out using multiple electronic databases, including ScienceDirect, Google Scholar, PubMed, and BASE. The process took place between July 2025 and December 2025. Besides peer-reviewed articles, relevant institutional reports, government documents, and policy publications were reviewed to gather updated information on vaccine production, vaccination programs, and livestock disease control in Bangladesh.

### Search terms

The literature search was performed using a mix of keywords and search strings related to veterinary vaccines and livestock health. The main search terms included: veterinary vaccines, vaccine production, livestock vaccines, disease control, animal health, vaccination coverage, livestock production, vaccine import dependency, veterinary vaccine development, and Bangladesh. These terms were used both alone and in various combinations with Boolean operators (AND, OR) to find relevant publications across different databases.

### Inclusion and exclusion criteria

Studies included in this review were those directly related to veterinary vaccines, livestock disease control, vaccine production systems, vaccination practices, or animal health management in Bangladesh. Publications on the epidemiology of major livestock diseases, vaccine development technologies, vaccination coverage, livestock production trends, and regulatory frameworks were also considered relevant. Only articles published in English from 2004 to 2025 were included, with a focus on studies published from 2020 onward to highlight recent developments in vaccine research and livestock health management. Articles unrelated to veterinary vaccines or livestock production, or written in languages other than English, were excluded from the review.

### Data extraction and synthesis

Initially, titles and abstracts of the identified studies were screened to assess their relevance. The full texts of selected articles were then reviewed, and relevant information was extracted. In total, about 65 scientific publications and institutional reports were included in this review. Data from these sources was organized and analyzed thematically to give a comprehensive overview of the veterinary vaccine sector in Bangladesh.

### Scope of the review

The reviewed literature was examined to explore several key aspects of veterinary vaccines in Bangladesh. These included the livestock population and disease burden, current vaccination practices, vaccine production and manufacturing systems, regulatory and institutional frameworks, import dependency, and emerging research and development initiatives. This comprehensive approach enabled the review to evaluate the current situation, identify major challenges, and highlight future opportunities for strengthening veterinary vaccine production and enhancing disease control strategies to support sustainable livestock production in Bangladesh.

## LIVESTOCK POPULATION AND ECONOMIC CONTRIBUTION

**Figure 1:**
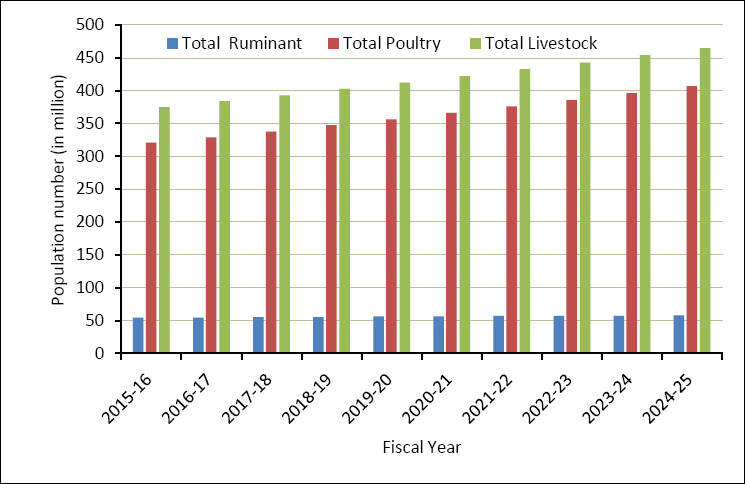
Livestock population trends in Bangladesh over the past decade [13].

The livestock sector is a vital part of Bangladesh’s agricultural economy, playing a major role in food security, rural livelihoods, and national economic growth [12]. Over the past decade, from fiscal year 2015–2016 to 2024–2025, livestock numbers have steadily increased (Figure 1) [13]. The poultry population, including chickens and ducks, saw the fastest growth, rising from about 320 million to over 406 million birds. Meanwhile, the ruminant population, which includes cattle, buffaloes, goats, and sheep, grew more slowly, from roughly 54 million to nearly 58 million animals. As a result, the overall livestock population grew from around 375 million to more than 464 million during this period [13]. These trends reflect ongoing growth in the livestock sector, mainly driven by the expansion of poultry production and steady improvements in ruminant farming systems.

The livestock sector also makes a significant contribution to the national economy. It accounts for approximately 1.81% of the gross domestic product (GDP) and contributes about 16.54% to agricultural GDP, with an annual growth rate of around 3.19% for the livestock sector [13]. Besides its economic importance, livestock plays a crucial role in rural employment. About 20% of the population is directly involved in livestock farming, while nearly 50% depend on livestock-related activities indirectly for their income and livelihoods [14].

**Figure 2:**
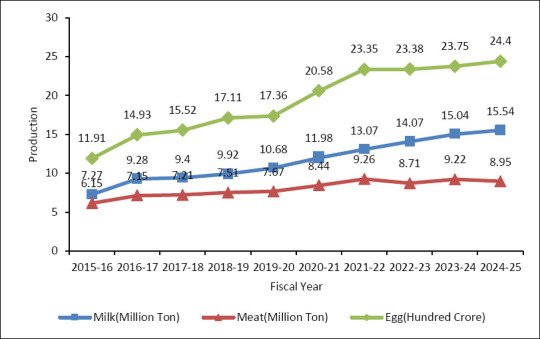
Trends in livestock production performance in Bangladesh [13].

Bangladesh’s livestock sector has also made significant progress in dairy and poultry production over the past decade. The output of milk, meat, and eggs has consistently grown from 2015–2016 to 2024–2025 (Figure 2) [13]. Milk production nearly doubled during this period, rising from 7.27 million tons to 15.54 million tons. Meat production increased moderately from 6.15 million tons to 8.95 million tons, though a slight decline was seen after 2021–2022. Egg production showed the most substantial growth, jumping from 11.91 hundred crore to 24.4 hundred crore eggs, with sharp increases particularly between 2019–2020 and 2021–2022 [15].

Overall, the steady rise in milk, meat, and egg production shows significant progress in the livestock sector. These gains are mainly due to better farm management, improved feeding systems, enhanced veterinary services, and more support from government and private sectors for dairy and poultry growth.

## REGIONAL HETEROGENEITY OF LIVESTOCK PRODUCTION

Livestock production in Bangladesh shows significant regional differences, with notable variations in the distribution of cattle, goats, sheep, and poultry across various administrative divisions due to agro-ecological, socio-economic, and geographic factors [16]. Divisional data reveal considerable differences in ruminant populations, from about 4.44 million in Sylhet and 5.34 million in Barishal to much higher numbers in Rangpur (17.88 million) and Rajshahi (17.24 million). Nationwide, commercial ruminant production makes up roughly 61.33% of the total population, though this percentage varies widely by region. For instance, the highest shares of commercial ruminants are found in Khulna (70.26%), Rajshahi (68.51%), and Dhaka (65.65%), while lower shares are recorded in Sylhet (42.01%) and Barishal (52.02%). Conversely, traditional livestock systems are more dominant in Sylhet (57.99%), Barishal (47.98%), and Mymensingh (45.13%) compared to Khulna (29.74%) and Rajshahi (31.49%) [17].

Similarly, the poultry sector also shows significant regional differences in both population size and production systems. Total poultry populations range from about 21.34 million in Sylhet to as many as 118.43 million in Dhaka. Other divisions, including Rajshahi (94.08 million) and Chattogram (86.46 million), have intermediate population sizes influenced by factors such as market demand, feed availability, and the level of poultry production systems. Nationally, commercial poultry production accounts for approximately 61.47% of the total poultry population; however, this share varies greatly among regions. Dhaka (76.31%) and Chattogram (67.92%) have the highest levels of commercialization, mainly driven by urban market demand and better infrastructure. In contrast, Rangpur has a relatively higher percentage of backyard poultry production (52.23%), highlighting the ongoing importance of traditional small-scale farming in rural areas. Divisions like Khulna, Barishal, and Sylhet display a more balanced mix of commercial and backyard poultry, while Mymensingh and Rajshahi lean more toward commercial systems. These regional differences highlight the need for tailored livestock development strategies and biosecurity measures across Bangladesh [17, 18].

## MAJOR INFECTIOUS DISEASES OF LIVESTOCK AND THEIR EFFECTS ON PRODUCTION

Livestock production in Bangladesh is often challenged by a wide variety of infectious diseases that seriously impact animal productivity and farmers’ livelihoods. Table 1 summarizes the most common and economically significant infectious diseases affecting livestock in Bangladesh and their effects on animal production [19, 20].

**Table 1 (A–C):** Major infectious diseases affecting livestock in Bangladesh and their impacts on production.

A. Cattle and buffalo			

Name of the disease	Causative agent	Prevalence and effect on animal production	Reference
Foot-and-mouth disease	*Aphthovirus*	Approximately 54.7% prevalence in sampled outbreaks; severe milk loss, reduced draught power, growth retardation, and trade restrictions	[21]
Peste des petits ruminants in large ruminants	*Morbillivirus*	In a particular study, the overall seroprevalence was 16% and 3.68% in sheep and cattle, respectively, whereas buffaloes exhibited a significantly higher seroprevalence of 42.36%	[22]
Hemorrhagic septicemia	*Pasteurella multocida* type B	Lower overall prevalence but distributed across many districts; sudden death and loss of draught animals	[23]
Anthrax	*Bacillus anthracis*	Lower overall prevalence and still represent an important bacterial disease burden; high mortality, zoonotic threat, and public panic	
Black quarter	*Clostridium chauvoei*	Sudden death in young cattle; investment loss	[24]
Lumpy skin disease	*Capripoxvirus*	Emerging outbreak pattern with notable morbidity in the affected regions; skin lesions, reduced milk yield, and hide damage	[25]

**B. Goat and sheep**			

**Name of the disease**	**Causative agent**	**Prevalence and effect on animal production**	**Reference**

Peste des petits ruminants	*Morbillivirus*	In goats, approximately 14.68% and in sheep, approximately 16% prevalence in examined small ruminants; high morbidity (80%–90%) and mortality (50%–90%): major cause of goat production loss	[26]
Contagious caprine pleuropneumonia	*Mycoplasma capricolum* subsp. *capripneumoniae*	Severe respiratory disease; high mortality rate	[24, 27]
Goat pox	*Capripoxvirus*	Reduced market value due to skin damage	

**C. Poultry**			

**Name of the disease**	**Causative agent**	**Prevalence and effect on animal production**	**Reference**

Newcastle disease	Avian paramyxovirus-1	The overall prevalence is approximately 13.33%; sudden high mortality and total flock loss	[28]
Avian influenza	Influenza A virus (H5 and H9)	High mortality, culling, zoonotic risk	
Infectious bursal disease (IBD)	Birnavirus	Prevalence is approximately 18.08%; immunosuppression, secondary infections	
Fowl cholera	*Pasteurella multocida*	In fowl, the prevalence of cholera is about 2.43%; high mortality in adults	

These diseases impact multiple livestock species and can cause significant economic losses through decreased productivity, higher mortality rates, and trade restrictions. In cattle and buffalo, diseases like FMD, PPR, HS, anthrax, black quarter, and LSD are particularly concerning because of their widespread spread and serious economic effects [21–25]. Likewise, small ruminants are often affected by diseases such as PPR, contagious caprine pleuropneumonia, and goat pox, which lead to high rates of illness and death and cause major losses in goat and sheep production systems [24, 26, 27].

In the poultry sector, diseases such as ND, AI, infectious bursal disease, fowl cholera, and mycoplasmosis pose significant challenges to both commercial and backyard poultry production in Bangladesh [28]. These diseases often cause rapid outbreaks, high mortality rates, reduced egg production, and increased expenses for disease control and biosecurity measures.

## IMPACT OF INFECTIOUS DISEASES ON LIVESTOCK PRODUCTION

Livestock diseases continue to be a major barrier to sustainable livestock production in Bangladesh, where the livestock sector accounts for about 1.9% of the country’s GDP. Over 10 million people depend either directly or indirectly on livestock for their livelihoods, making up nearly 16% of the population [29]. The effects of livestock diseases are wide-ranging, impacting economic stability, food security, and public health.

Economically, outbreaks of diseases such as FMD [30], PPR, HS, anthrax, LSD, ND, and AI lead to significant direct losses from increased mortality and decreased production of milk, meat, and eggs. For instance, the annual economic loss from FMD outbreaks is estimated to be about 2.22 billion USD [31]. Likewise, PPR outbreaks result in considerable financial losses for smallholder farmers [32], while HS alone can cause annual economic losses of roughly 24.56 million USD and 14.74 million USD in affected regions [32].

Indirect effects of infectious diseases worsen these losses by causing reduced fertility, growth delays, and longer recovery times in affected animals, which lowers lifetime productivity. Disease outbreaks can also lead to market disruptions, including trade restrictions related to diseases such as LSD, AI, and FMD, further increasing the economic burden on farmers and livestock producers [34, 35]. Additionally, expenses for treatment, vaccination, and biosecurity measures raise production costs, especially for smallholder farmers.

Livestock diseases also impact food security and public health. Declines in animal production decrease the availability of affordable animal-source proteins, which can lead to nutritional deficiencies, especially in rural communities. Additionally, several livestock diseases are zoonotic and pose direct risks to human health. Zoonotic infections such as rabies, AI, anthrax, and brucellosis can cause serious public health issues and often create consumer anxiety, reducing demand for animal-origin foods [36]. Rabies remains the top priority among zoonotic diseases due to its endemic nature and almost 100% fatality rate after symptoms appear [37]. AI is also considered a major zoonotic threat because of its pandemic potential and frequent transmission from poultry to humans. Outbreaks of anthrax continue to affect both livestock and people, particularly those with occupational exposure to animals. Brucellosis also presents a significant zoonotic concern because of its long-term health effects and economic impact.

Overall, the cumulative impacts of infectious livestock diseases not only reduce farmer income and rural employment but also limit the growth potential of the livestock sector, which is essential for meeting the rising national demand for animal-source food and supporting sustainable livelihoods.

## TRENDS IN VETERINARY VACCINE USAGE IN LIVESTOCK

Research conducted in Bangladesh consistently shows low compliance with veterinary vaccination programs due to knowledge gaps and practical obstacles faced by farmers. For instance, only 59.4% of large ruminant farmers reported vaccinating their herds, while just 27.8% demonstrated proper vaccination practices despite 73.8% being aware of vaccines [38]. Moreover, many farmers lacked sufficient understanding of the preventive role of vaccines and often failed to check vaccine expiry dates, revealing weaknesses in vaccine handling and management practices.

Similarly, about 61.8% of small ruminant farmers reported vaccinating their animals; however, 59.6% did not follow recommended vaccination schedules, and 56.6% failed to keep immunization records. Only 22.8% of farmers demonstrated proper vaccination practices, highlighting significant gaps in small ruminant health management [39].

In the poultry sector, vaccination coverage seems relatively high; about 70.8% of poultry farmers reported vaccinating their flocks. However, only 37.3% followed recommended vaccination schedules, and many farmers did not keep vaccination records or store vaccines properly. Additionally, 25.4% of farmers reported the unavailability of certain vaccines, which further leads to missed vaccinations and inconsistent disease prevention practices [39].

A national study on anthrax vaccination programs also identified operational challenges in conducting the campaigns. Mass vaccination efforts reached only about 44% of cattle, and more than half of the farmers reported difficulty handling cattle as a major obstacle to participating in the vaccination programs [40].

Despite these challenges, the overall use of veterinary vaccines in Bangladesh’s livestock sector has gradually grown, although vaccination coverage still varies across different production systems. Coverage is generally highest in commercial poultry farms, where vaccination is routinely part of intensive production practices. Conversely, vaccination coverage in backyard poultry systems remains inconsistent due to limited awareness, logistical challenges, and restricted access to veterinary services.

Vaccination coverage for major livestock diseases, including PPR, FMD, anthrax, ND, and AI, has increased in recent years due to routine vaccination programs and targeted mass campaigns. Large-scale PPR vaccination efforts, in particular, have reached high coverage levels in small ruminants like goats and sheep, with some areas reporting vaccination rates over 90%.

In cattle populations, vaccination coverage against diseases like anthrax, FMD, and LSD remains relatively limited. For example, anthrax vaccination coverage typically ranges from 25% to 40%, depending on the specific campaign and location (Table 2) [41–45]. However, comprehensive nationwide long-term data on vaccination coverage for each disease are still scarce. Most available information comes from campaign reports, vaccine distribution records, and regional studies rather than systematic nationwide surveillance.

**Table 2:** Veterinary vaccine usage pattern in Bangladesh’s livestock sector.

Species	Usage pattern	Source
Commercial poultry	High (i.e., routine vaccination is standard practice in commercial farms)	[41]
Backyard poultry	Low and variable (many small flocks remain unvaccinated)	[42]
Goats and sheep (Peste des petits ruminants)	Very high in mass campaign zones (>90%); low routine coverage in other areas	[43]
Cattle (Anthrax, foot-and-mouth disease, black quarter, hemorrhagic septicemia, etc.)	A study involving 733 farmers revealed that only 62% vaccinated their animals against *Bacillus anthracis*, *Aphthovirus*, *Clostridium chauvoei*, or *Pasteurella multocida* type B. Among the vaccinating farmers, 26% administered the anthrax vaccine exclusively, whereas 68% administered it in combination with at least one other vaccine	[44]
Dogs (rabies mass dog vaccination campaigns)	Mass dog vaccination programs reported substantial numbers per district (e.g., 21,000 dogs/district/year in some programs)	[45]

## REGULATORY FRAMEWORK FOR VETERINARY VACCINES

The regulatory oversight of veterinary vaccines in Bangladesh involves multiple government agencies responsible for ensuring the safety, effectiveness, and quality of veterinary biological products. The Directorate General of Drug Administration (DGDA), under the Ministry of Health and Family Welfare, serves as the country’s main regulatory body responsible for evaluating and approving the registration of veterinary vaccines. This process guarantees that both imported and domestically produced vaccines meet established safety, effectiveness, and quality standards before they are authorized for distribution and use within the country.

The DLS, under the Ministry of Fisheries and Livestock, is the main agency responsible for carrying out livestock health programs. The DLS manages national vaccination campaigns, disease surveillance, and vaccine distribution through its wide network of district and upazila veterinary offices.

The LRI is a key player in producing and developing veterinary vaccines in Bangladesh. As a public-sector facility dedicated to production and research, LRI manufactures vital vaccines for livestock and poultry diseases and supplies them to the DLS for nationwide immunization efforts.

Additionally, the Bangladesh Livestock Research Institute (BLRI) makes important contributions to veterinary vaccine research and development. BLRI conducts advanced studies on disease diagnostics, pathogen identification, and vaccine creation, including developing new vaccine candidates for emerging and endemic livestock diseases. The institute also works with the DLS to transfer vaccine seed strains and support field-level vaccine application.

## GOVERNMENT POLICIES AND INITIATIVES FOR VETERINARY VACCINES

The Government of Bangladesh has implemented several strategic measures to boost veterinary vaccine production, improve disease management, and promote livestock health. These efforts aim to decrease economic losses caused by transboundary and endemic animal diseases and to strengthen national food security. Key government initiatives include the following:

### Local vaccine development

Efforts are underway to expand domestic vaccine production for key livestock diseases such as FMD and LSD. These initiatives aim to reduce dependence on imported vaccines while enhancing disease control through locally adapted vaccine formulations [46].

### Institutional strengthening

BLRI plays a key role in vaccine research and development, including work on vaccines for LSD, AI, and goat pox as part of broader efforts to address zoonotic and transboundary animal diseases [47, 48]. Meanwhile, the DGDA has introduced regulatory guidelines for veterinary vaccine registration and mandates that vaccine manufacturers comply with GMP to ensure product safety, efficacy, and affordability [49].

### Mass vaccination campaigns

Large-scale vaccination initiatives have been carried out under national development programs such as the National Agricultural Technology Program Phase II and the Livestock and Dairy Development Project. These programs, supported by international partners like the World Bank and the International Fund for Agricultural Development, promote nationwide vaccination campaigns, farmer training programs, and improvements in vaccine distribution systems, thereby benefiting millions of livestock and poultry [50].

### Rabies eradication under One Health

Bangladesh has launched a National Rabies Elimination Program based on the One Health approach. The program combines human and animal health efforts, including mass dog vaccination campaigns and public awareness initiatives, which have greatly decreased human rabies deaths in recent years [51].

### Biosafety and biosecurity measures

Biosafety and biosecurity regulations related to veterinary vaccines in Bangladesh are mainly included within broader laws that govern animal health and infectious disease control. These rules focus on controlling pathogens, quarantine procedures, and disease prevention at farms, labs, and animal markets. These policies support the country’s commitments to biosafety and biosecurity as part of larger public health security efforts. However, the enforcement of these measures, especially at the farm level and during vaccine storage and distribution, is inconsistent and needs improvement through better training, stricter regulation, and standardized operational procedures.

## HISTORY AND DEVELOPMENT OF VETERINARY VACCINE PRODUCTION IN BANGLADESH

The development of veterinary vaccine production in Bangladesh has gradually advanced from small-scale government efforts to a more diverse system involving both public and private institutions. Early vaccine production activities were fairly limited and mainly focused on a few traditional vaccines, with little documentation of production volumes or efficiency metrics. As the livestock sector grew and disease control became more critical, vaccine production capacity steadily increased under government institutions. However, detailed historical comparisons of vaccine productivity, manufacturing efficiency, and cost-effectiveness are still poorly documented.

### Early efforts in vaccine production

The LRI, operated by the Department of Livestock Services, was founded in 1956 as Bangladesh’s first national institute dedicated to veterinary vaccine production. The institute was established to support disease control efforts and safeguard the livestock industry from economically important infectious diseases.

During the early decades after its founding, LRI mainly focused on producing conventional bacterial and viral vaccines for livestock and poultry diseases. The earliest vaccines included those for anthrax, BQ, HS, fowl cholera, and ranikhet disease, including the baby chick ranikhet disease vaccine used in poultry systems [52]. These vaccines played a crucial role in early national disease control efforts and helped improve livestock health and productivity.

### Evolution of vaccine manufacturing facilities

Over time, Bangladesh’s vaccine manufacturing sector has shifted from being mainly government-controlled to a mixed public–private system. Initially, the government, through LRI, almost solely managed vaccine production, creating essential vaccines for key livestock diseases. These included vaccines for anthrax, FMD, LSD, HS, BQ, PPR, goat pox, ND, duck plague, salmonella infections, fowl cholera, fowl pox, gumboro disease, and Marek’s disease [53].

In recent decades, the sector has expanded with the emergence of private pharmaceutical companies that contribute to veterinary vaccine production. Since the late 2000s, companies such as Incepta and FnF Pharmaceuticals have started establishing modern production facilities that include upstream antigen manufacturing and high-throughput fill-finish technologies. Initially, these companies focused mainly on poultry vaccines due to the rapid growth of the poultry industry. Over time, however, private manufacturers have gradually broadened their production portfolios to include vaccines targeting ruminant diseases.

The introduction of GMP in several production facilities, enhancements in quality control systems, and expansion of cold-chain infrastructure have further strengthened the national vaccine production ecosystem.

### Technological advancements in veterinary vaccine development

Technological progress in veterinary vaccine development in Bangladesh has been promising but uneven. Research efforts have increasingly centered on recombinant and multiepitope vaccine candidates targeting locally prevalent pathogens. However, many of these projects remain fragmented and are not yet part of a coordinated national innovation framework.

Several academic and research institutions are currently developing polyvalent and pathogen-specific vaccines, including candidates targeting diseases such as mastitis. However, the progress of these vaccine candidates from laboratory research to large-scale industrial production remains limited. Notably, formal assessments of technology readiness levels for vaccine candidates are rarely documented, making it hard to track the transition from research prototypes to regulatory approval and field deployment.

Scaling vaccine production from pilot-scale batches made in universities or research labs to full-scale industrial manufacturing that meets GMP standards remains a major challenge. These issues are partly due to infrastructure limitations, regulatory gaps, and a lack of coordination among research institutions, regulatory agencies, and industry partners. Despite these obstacles, Bangladesh has started incorporating modern biotechnological methods, such as genomic surveillance, locally adapted vaccine design, and emerging digital technologies, into veterinary vaccine research. These advances offer a hopeful foundation for future progress in precision vaccine development.

#### Genome-informed vaccine development

Recent genomic studies have enhanced the understanding of locally circulating viral strains. Researchers performed whole-genome sequencing of PPR virus isolates collected in Bangladesh from 2008 to 2020 and identified conserved epitopes shared with existing vaccine strains. These findings suggest the potential for developing locally adapted PPR vaccines based on indigenous viral strains [54].

#### Locally developed vaccine for FMD

Researchers at Dhaka University successfully decoded the genomes of three circulating serotypes of the FMD virus (O, A, and Asia 1) detected in Bangladesh between 2012 and 2018. Based on these findings, they developed a trivalent FMD vaccine aimed at providing broader protection against the circulating viral strains in the country [55, 56].

#### Vaccination initiatives for AI

Bangladesh launched pilot vaccination programs against AI during 2012–2013, utilizing several innovative vaccine formulations, including Vectormune HVT-AI (Ceva), Re6, and Nobilis H5 inactivated vaccines. Later, an H9N2 vaccine was approved and showed promising antibody responses in vaccinated poultry flocks [57].

#### Local production of the LSD vaccine

A major milestone in domestic vaccine development happened in early 2025, when the BLRI provided the initial LSD vaccine seed strain to the DLS for field use. This step marks an important move toward boosting domestic vaccine production and could open up future opportunities for vaccine exports [58].

#### Biotechnology and infrastructure contributions

Bangladesh Agricultural University has also contributed to national vaccine development initiatives by providing vaccine seed strains to the DLS in 2024 [59]. Additionally, national policy frameworks such as the National Biotechnology Policy-2012, along with institutional support from organizations including the National Institute of Biotechnology, have laid the groundwork for the future development of veterinary vaccine technologies in Bangladesh.

## CURRENT STATUS OF VETERINARY VACCINES AND VACCINATION IN BANGLADESH

### Major institutions and manufacturers of veterinary vaccines

In Bangladesh, veterinary vaccine production and supply involve a mix of public-sector agencies and private pharmaceutical firms. However, available information indicates that the vaccine market remains uneven, with limited detailed data on production capacities and market distribution.

#### Livestock Research Institute

The LRI is the main government agency responsible for veterinary vaccine production, research, and distribution in Bangladesh. It plays a vital role in supporting the nation’s immunization efforts to control major livestock and poultry diseases. LRI produces a wide variety of traditional and modern veterinary vaccines and has been a key part of the country’s vaccine supply system for many years [60].

#### BLRI

The BLRI operates as an autonomous research agency under the Government of Bangladesh and plays a key role in advancing veterinary vaccine research and development. BLRI makes important contributions to disease diagnostics, epidemiological studies, and vaccine development for both emerging and endemic livestock diseases. Besides conducting research, the institute works with the DLS to develop vaccine seed strains and support their implementation in the field.

#### Private pharmaceutical companies

Over the last twenty years, private pharmaceutical companies have greatly increased their capacity to produce veterinary vaccines in Bangladesh. Companies like Incepta Pharmaceuticals and FnF Pharmaceuticals are actively engaged in manufacturing veterinary vaccines, mainly for the poultry industry. Additionally, multinational pharmaceutical firms participate in the national vaccine market through importation and technical collaborations, offering access to internationally developed veterinary vaccines.

### Types of veterinary vaccines produced locally

A variety of veterinary vaccines are produced locally in Bangladesh to protect livestock and poultry from major infectious diseases. These vaccines target a broad range of bacterial and viral pathogens affecting cattle, small ruminants, poultry, and companion animals. The main vaccines produced domestically, along with their target species, vaccine type, storage requirements, shelf life, and manufacturers, are summarized in Table 3 [53].

**Table 3:** Types of locally produced veterinary vaccines in Bangladesh [53].

Disease	Animal(s) targeted	Type of vaccine	Typical cold-chain storage temperature	Shelf life (unopened)	Producers
Anthrax	Cattle, goats, and sheep	Live spore vaccine	2°C to 8°C	6 months	LRI
Hemorrhagic septicemia	Cattle, buffalo	Inactivated (bacterin)	2°C to 8°C	6 months	LRI
Black quarter	Cattle, buffalo	Inactivated (bacterin)	2°C to 8°C	6 months	LRI
Peste des petits ruminants	Goats and sheep	Live attenuated	2°C to 8°C or –20°C	6 months (2°C to 8°C); 2 years (–20°C)	LRI
Foot-and-mouth disease	Cattle, buffalo, and goats	Inactivated (oil-adjuvanted)	2°C to 8°C	6 months	LRI, FnF Pharma, Incepta
Goat pox	Sheep, goats	Inactivated (bacterin)	2°C to 8°C or –20°C	6 months (2°C to 8°C); 1 year (–20°C)	LRI
Lumpy skin disease	Cattle, buffalo	Inactivated (bacterin)	2°C to 8°C or –20°C	6 months (2°C to 8°C); 1 year (–20°C)	LRI, BLRI
Rabies	Dogs, cattle	Inactivated (cell culture)	2°C to 8°C	6 months	Incepta, FnF
Newcastle disease – BCRDV and RDV	Poultry (chickens and ducks)	Live attenuated and inactivated	2°C to 8°C or –20°C	4 months (2°C to 8°C); 1 year (–20°C)	LRI, Incepta, FnF
Avian influenza (AI-H5N1 and H9N2)	Poultry	Inactivated	2°C to 8°C	6 months	Incepta, FnF
Infectious bursal disease	Poultry	Live and inactivated	–20°C to 0°C	1 year	LRI, Incepta
Fowl pox	Poultry	Live attenuated	–5°C to 0°C or –20°C	6 months (–5°C to 0°C); 1 year (–20°C)	LRI, Incepta
Pigeon pox	Pigeons	Live attenuated	–5°C to 0°C or –20°C	6 months (–5°C to 0°C); 1 year (–20°C)	Not specified
Marek’s disease	Poultry	Live (cell culture–based)	–20°C	1 year	LRI, Incepta
Duck plague	Ducks	Live attenuated	–5°C to 0°C	6 months	LRI
Fowl cholera	Poultry	Inactivated	2°C to 8°C	6 months	LRI
Salmonellosis	Poultry	Live (cell culture–based)	2°C to 8°C	6 months	LRI

BLRI = Bangladesh Livestock Research Institute, LRI = Livestock Research Institute.

Domestic vaccine production mainly targets diseases like anthrax, HS, BQ, PPR, FMD, LSD, ND, AI, and several poultry diseases such as infectious bursal disease, fowl pox, Marek’s disease, duck plague, and fowl cholera. These vaccines are produced by public institutions like LRI and BLRI, as well as private pharmaceutical companies including Incepta and FnF Pharmaceuticals. Proper storage conditions, usually between 2°C and 8°C or −20°C depending on the vaccine type, are essential to maintain vaccine effectiveness and stability.

### Vaccine production trends by LRI over the past decade

**Figure 3:**
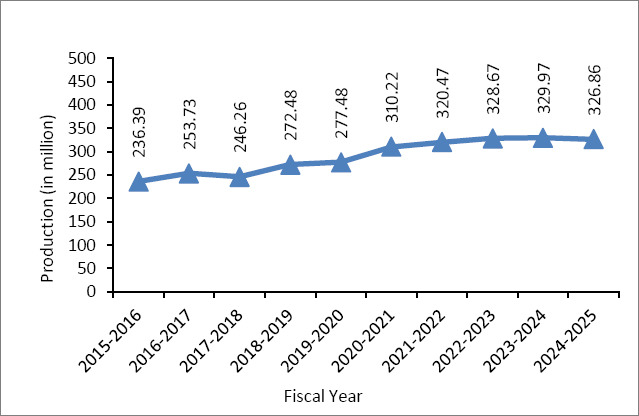
Vaccine production trends (million doses) by Livestock Research Institute during the past decade [53].

Over the past decade, vaccine production at LRI has significantly increased in response to the rising demands of the livestock sector. In fiscal year 2015–2016, LRI produced about 236 million doses of vaccines for cattle, goats, sheep, and poultry. Continuous improvements in production infrastructure and manufacturing capacity allowed a steady rise in output, reaching around 327 million doses by fiscal year 2024–2025 (Figure 3) [53]. This growth reflects an average annual increase of approximately 3%–4%.

In recent years, vaccine production at LRI has stabilized around this capacity level, providing a relatively steady supply of vaccines for major livestock and poultry diseases. The institute remains a vital part of the national animal health system and makes significant contributions to disease prevention and food security.

Detailed production data from the past three fiscal years further demonstrate the trends in vaccine production by both public and private manufacturers (Table 4) [53]. These data highlight significant differences between ruminant and poultry vaccine production.

**Table 4:** Vaccine production by the Livestock Research Institute and private manufacturers in Bangladesh (2022–2025) [53].

Fiscal year	Livestock Research Institute		Private manufacturers	

	Ruminant vaccines (million doses)	Poultry vaccines (million doses)	Ruminant vaccines (million doses)	Poultry vaccines (million doses)
2022–2023	27.01	301.67	1.48	1469.56
2023–2024	17.99	311.97	1.95	1918.78
2024–2025	19.22	306.19	2.50	2411.71

Between 2022–2023 and 2024–2025, ruminant vaccine production at LRI fluctuated, decreasing from 27.01 million doses in 2022–2023 to 17.99 million doses in 2023–2024, then slightly rising to 19.22 million doses in 2024–2025. Meanwhile, poultry vaccine production at LRI stayed relatively steady at around 300 million doses each year.

Private manufacturers, however, showed a different production pattern. Their ruminant vaccine production remained relatively small but steadily grew from 1.48 million doses to 2.50 million doses during the same period. Poultry vaccine production by private manufacturers increased significantly, jumping from 1469.56 million doses in 2022–2023 to 2411.71 million doses in 2024–2025. These trends indicate that while LRI maintains steady production for national immunization programs, private manufacturers have quickly expanded their role in supplying poultry vaccines and now dominate this part of the market.

### Vaccination status of DLS vaccines provided by LRI

**Figure 4:**
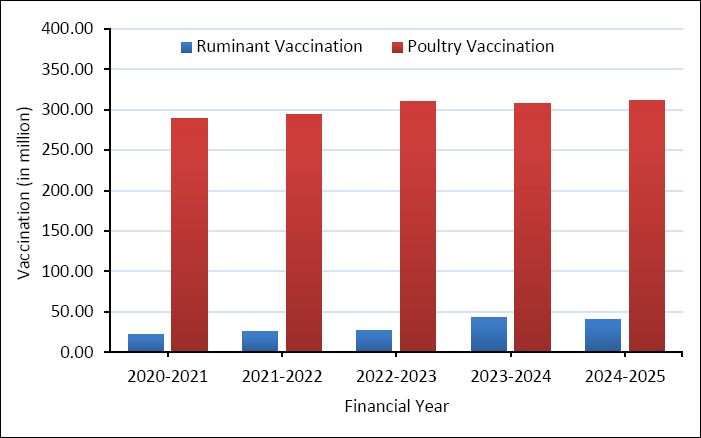
Vaccination coverage in livestock and poultry by Department of Livestock Services during the past five years [61].

Vaccination programs carried out by the DLS have shown steady progress in recent years. Ruminant vaccination coverage grew from about 20 million doses administered in 2020–2021 to nearly 45 million doses in 2023–2024, before slightly decreasing in 2024–2025 (Figure 4) [61]. This overall trend shows improved vaccination rates among ruminant populations, although it still remains significantly lower than in poultry.

In contrast, poultry vaccination coverage has stayed consistently high, with annual doses ranging from about 290 to 310 million. This stability shows the strong demand for vaccination in commercial poultry production and the efforts of both public and private sector vaccine providers.

Overall, the comparison between vaccine production and vaccination coverage shows that poultry vaccination programs in Bangladesh are fairly well established, while ruminant vaccination coverage remains comparatively limited. The rapid growth of poultry vaccine production by private manufacturers has been crucial in maintaining high vaccination coverage in the poultry sector.

### Quality control and regulatory framework for veterinary vaccines

Bangladesh’s regulation of veterinary vaccines aims to ensure their safety, effectiveness, and quality before they are used in livestock and poultry. The DLS, under the Ministry of Fisheries and Livestock, acts as the main authority responsible for overseeing vaccine production, distribution, and implementation in the field.

Veterinary vaccines are produced locally by LRI and related vaccine manufacturing facilities, as well as by some private pharmaceutical companies. Imported vaccines must undergo regulatory approval processes conducted jointly by the DGDA and the DLS [62].

Quality control activities are carried out by the Vaccine Production Unit and the Quality Control Section of LRI. These units perform standard laboratory evaluations, including sterility testing, safety testing, potency assessment, and purity analysis, following guidelines established by international organizations such as the World Organization for Animal Health and the World Health Organization.

The regulatory system also involves inspecting production facilities, conducting batch-release testing, monitoring compliance with GMP, overseeing pharmacovigilance for adverse vaccine reactions, maintaining cold-chain systems, and performing field-level monitoring of vaccine performance. Despite these regulatory measures, publicly available evidence about independent third-party quality audits of veterinary vaccine manufacturers and DGDA laboratories remains limited.

## VACCINE IMPORTATION AND DEPENDENCE

### Imported veterinary vaccines and their volumes

Bangladesh largely depends on imported veterinary vaccines to meet the health needs of ruminants, poultry, and pet animals. Domestic production, mainly carried out by the LRI and a few private manufacturers, accounts for only a small part of the national demand, while the rest is primarily satisfied through imports [63]. The main veterinary vaccines imported into Bangladesh for ruminants, poultry, and pet animals are summarized in Table 5 [64].

**Table 5:** Commonly imported veterinary vaccines in Bangladesh [64].

Animal category	Imported vaccine	Target disease
Ruminant	Foot-and-mouth disease and peste des petits ruminants vaccines	Foot-and-mouth disease and peste des petits ruminants
Poultry	Infectious bursal disease/Gumboro vaccine	Infectious bursal disease (also known as Gumboro disease)
Poultry	Newcastle disease vaccine	Newcastle disease
Poultry	Avian influenza vaccine	Avian influenza
Poultry	Infectious bronchitis vaccine	Infectious bronchitis
Poultry	Fowl cholera vaccine	Fowl cholera
Poultry	Infectious coryza vaccine	Infectious coryza
Poultry	*Mycoplasma gallisepticum* vaccine	*Mycoplasma gallisepticum* disease
Poultry	Marek’s disease vaccine	Marek’s disease
Poultry	Combined poultry vaccine	Combined poultry pathogens
Pet animal	Rabies vaccine	Rabies
Pet animal	FVRCP vaccine	Feline viral rhinotracheitis, calicivirus, and panleukopenia (commonly referred to as cat flu)

The volume of imported veterinary vaccines has fluctuated over the past 3 fiscal years (Table 6) [65]. While import volumes for ruminant and poultry vaccines varied during this period, imports of pet animal vaccines consistently increased, reflecting a growing diversification in national vaccine demand across different animal sectors. Overall, these data highlight Bangladesh’s strategic reliance on international vaccine sources to support livestock and companion animal health.

**Table 6:** Imported veterinary vaccines in Bangladesh over the past 3 fiscal years [65].

Fiscal year	Vaccine type	Quantity (doses)	Country of origin
July 2022–June 2023	Ruminant	77.75 million	Russia, India, Nigeria, and the Netherlands
	Poultry	12.11 billion	China, Hungary, India, the Netherlands, Italy, Russia, Germany
	Pet animal	47.00 thousand	Korea, Netherlands, Italy, Germany
July 2023–June 2024	Ruminant	56.83 million	Russia, India, Nigeria, and the Netherlands
	Poultry	35.30 billion	China, United States, Hungary, India, Netherlands, Italy, Russia, Germany, France
	Pet animal	62.50 thousand	Korea, Netherlands, Italy, Germany
July 2024–June 2025	Ruminant	73.54 million	Russia, India, Nigeria, and the Netherlands
	Poultry	43.66 billion	China, United States, Hungary, India, Netherlands, Italy, Russia, Germany, France
	Pet animal	1.47 million	Korea, Netherlands, Italy, Germany

Imports of ruminant vaccines dropped from 77.75 million doses in fiscal year 2022–2023 to 56.83 million doses in fiscal year 2023–2024, then increased to 73.54 million doses in fiscal year 2024–2025. This fluctuation might reflect changes in disease prevalence, campaign efforts, and the limited but variable role of domestic production. Major supplier countries for ruminant vaccines included Russia, India, Nigeria, and the Netherlands.

In contrast, imports of poultry vaccines increased significantly during the same period. Imported poultry vaccines grew from 12.11 billion doses in fiscal year 2022–2023 to 35.30 billion doses in fiscal year 2023–2024, and further rose to 43.66 billion doses in fiscal year 2024–2025 (Table 6) [65]. This upward trend indicates the rapid growth of Bangladesh’s poultry industry and the increasing focus on biosecurity and disease prevention, especially for AI, ND, and other economically important viral diseases. Poultry vaccines were imported from various countries, including China, the United States, Hungary, India, the Netherlands, Italy, Russia, Germany, and France.

Pet animal vaccine imports accounted for a much smaller share of total vaccine imports; however, they increased steadily from 47.00 thousand doses in fiscal year 2022–2023 to 62.50 thousand doses in fiscal year 2023–2024, then rose sharply to 1.47 million doses in fiscal year 2024–2025. This increase likely reflects growing pet ownership and heightened awareness of zoonotic disease prevention in urban areas. Korea, the Netherlands, Italy, and Germany were the principal suppliers of pet vaccines.

### Demand fulfillment by locally produced vaccines

The relationship between domestic vaccine production and national demand in Bangladesh is marked by significant uncertainty due to variability in data sources, estimation methods, and long-term planning. The methods used to estimate vaccine demand are rarely described in enough detail in either official reports or academic literature. Available estimates are mostly based on livestock and poultry population figures and often do not clearly consider herd structure, replacement rates, disease prevalence, vaccination schedules, or differences in production systems. This reduces the accuracy of national demand assessments.

Moreover, despite the rapid growth of commercial poultry farming and the changing epidemiological risks of infectious diseases, publicly available demand forecasting models for the next 5–10 years remain limited. The lack of such projections hinders effective planning for production growth, import needs, and strategic vaccine stock management.

Although Bangladesh produces veterinary vaccines for both ruminants and poultry, domestic output remains significantly below the national demand, resulting in ongoing reliance on imports. During the past three fiscal years, local production has met only a small part of the vaccine needs in both sectors (Table 7) [53, 65].

**Table 7:** Demand fulfillment by locally produced vaccines [53, 65].

Fiscal year	Total ruminant vaccine production (million doses)	Total imported ruminant vaccines (million doses)	Demand fulfillment by local vaccines (%)	Total poultry vaccine production (billion doses)	Total imported poultry vaccines (billion doses)	Demand fulfillment by local vaccines (%)
2022–2023	28.49	77.75	26.82	1.77	12.11	12.75
2023–2024	19.94	56.83	25.97	2.23	35.30	5.94
2024–2025	21.72	73.54	22.80	2.72	43.66	5.86

Ruminant vaccine production experienced moderate fluctuations, with total domestically produced doses dropping from 28.49 million in fiscal year 2022–2023 to 19.94 million in 2023–2024, then slightly increasing to 21.72 million in 2024–2025. Meanwhile, ruminant vaccine imports consistently remained much higher than domestic output each year. As a result, the share of ruminant vaccine demand met by domestic production decreased from 26.82% in 2022–2023 to 22.80% in 2024–2025.

For poultry vaccines, local production increased steadily from 1.77 billion doses in fiscal year 2022–2023 to 2.72 billion doses in fiscal year 2024–2025. However, this growth was greatly overshadowed by imports, which surged from 12.11 billion doses to 43.66 billion doses during the same period. As a result, the share of poultry vaccine demand met by domestic production decreased from 12.75% in fiscal year 2022–2023 to just 5.86% in fiscal year 2024–2025.

Overall, these data show that domestically produced ruminant vaccines currently meet about 23%–27% of the national demand, while locally produced poultry vaccines make up only around 6%–13% of the total demand (Table 7) [53, 65]. These findings clearly illustrate Bangladesh’s growing reliance on imported vaccines and emphasize the urgent need for increased manufacturing capacity, better production infrastructure, and supportive policies to boost domestic vaccine self-reliance.

### Reasons for import dependency

The livestock and poultry sectors in Bangladesh are growing quickly, leading to higher demand for vaccines against major infectious diseases. However, domestic vaccine production, mainly driven by LRI and a few private companies, still only meets a small part of the country’s needs. As a result, Bangladesh remains heavily reliant on imported vaccines.

Another significant limitation is that available import data are usually presented in dose volume only and rarely include price or value information. Consequently, the true extent of foreign exchange expenditure and the economic vulnerability linked to vaccine import dependence remains insufficiently measured. Additionally, there is little evidence of structured national risk assessments addressing potential vaccine supply disruptions caused by geopolitical instability, export restrictions, trade barriers, or global logistics interruptions. Such vulnerabilities could jeopardize routine vaccination programs and disease control efforts.

Several key factors contribute to this import dependency.

### Limited domestic production capacity

LRI and a few private manufacturers currently produce only a small portion of the vaccines needed for national livestock and poultry health programs. Existing production facilities are not yet able to meet the large and quickly growing demand across various animal sectors.

### Technological and infrastructure gaps

Advanced vaccine technologies, including recombinant, inactivated, and multivalent vaccine platforms, are not yet widely adopted in Bangladesh. Furthermore, many production facilities still rely on older equipment and manufacturing systems with limited scalability for industrial growth.

### High demand from expanding livestock and poultry sectors

The rapid expansion of commercial poultry, dairy, and other intensive livestock systems has increased the demand for a wider variety of veterinary vaccines. Domestic manufacturers have struggled to keep up with both the growing volume and diversity of vaccine needs.

### Disease variability and strain diversity

Frequent outbreaks of transboundary and emerging animal diseases like FMD, LSD, and AI demand vaccines that match locally circulating strains. Imported vaccines are often preferred because they are seen to offer broader or more reliable coverage against globally relevant strains.

### Quality assurance and reliability concerns

Commercial farms and field veterinarians often prefer imported products because of their proven performance, brand recognition, and perceived reliability. Limitations in post-production quality assessment and independent benchmarking of domestic vaccines may lessen confidence in locally made products.

### Regulatory and policy constraints

Lengthy approval and registration procedures can delay the development and commercialization of new domestic vaccines. Additionally, stronger government incentives are necessary to promote large-scale vaccine research and development and to encourage industrial investment in local production.

### Economic factors

In many cases, importing vaccines is quicker and may be more cost-effective than building advanced local manufacturing capacity. Additionally, multinational pharmaceutical companies hold significant influence over product availability, market preferences, and distribution channels in the veterinary pharmaceutical sector.

## PROSPECTS AND OPPORTUNITIES IN VETERINARY VACCINE PRODUCTION

Veterinary vaccination remains one of the most cost-effective strategies for controlling infectious diseases in livestock and poultry. Although Bangladesh produces several vaccines locally through the LRI and a limited number of private manufacturers, the country still depends heavily on imported vaccines to meet national demand. Enhancing domestic vaccine research, development, and production capacity is therefore crucial for improving national disease control programs and ensuring long-term livestock health security.

### Strengths

Several structural advantages support the development of veterinary vaccine production in Bangladesh.

- The presence of established public-sector vaccine production infrastructure, especially the LRI and its associated laboratories, which have extensive experience in manufacturing traditional bacterial and viral vaccines.
- National vaccination programs targeting major transboundary diseases such as FMD, PPR, ND, and AI, which create consistent baseline demand for veterinary vaccines.
- Growing policy and government recognition of the livestock and poultry sectors as vital contributors to food security, rural employment, and economic growth.

These strengths offer a crucial foundation for increasing domestic vaccine production and enhancing disease control efforts.

### Weaknesses

Despite advancements in veterinary vaccine manufacturing, Bangladesh still faces several structural barriers that limit self-sufficiency and quality assurance.

#### Limitations in technology and infrastructure

Many vaccine production facilities still depend on traditional manufacturing methods with limited automation and outdated equipment. Advanced vaccine technologies, including recombinant, subunit, and multivalent vaccine platforms, remain underdeveloped in the country. As a result, production scalability and product diversity are limited.

#### Strain adaptation challenges

Frequent outbreaks of transboundary diseases like FMD, ND, and AI require vaccines that closely match locally circulating strains. However, the lack of ongoing strain surveillance and regular vaccine updates can decrease vaccine effectiveness and hinder disease control efforts.

#### Shortage of skilled manpower

Veterinary vaccine production requires multidisciplinary expertise involving virologists, molecular biologists, immunologists, and skilled laboratory technicians. The availability of highly trained personnel remains limited compared to the increasing demands of vaccine research, production, and quality assurance.

#### Regulatory and policy barriers

Lengthy regulatory approval processes, overlapping responsibilities among regulatory authorities, and weak monitoring systems can delay the innovation and commercialization of new vaccines. Additionally, policies that support collaboration between public research institutions and private manufacturers are still not well-defined.

#### Limited research and development investment

Investment in applied research and product-focused vaccine innovation stays relatively low. Many domestic vaccine projects rely on imported master seed strains, which restricts the growth of local vaccine technologies.

#### Economic and market challenges

Domestic vaccine production often faces high manufacturing costs because of reliance on imported raw materials such as adjuvants, stabilizers, and cell culture media. Additionally, competition from cheaper imported vaccines can diminish the market competitiveness of locally produced vaccines.

#### Cold-chain and distribution constraints

Maintaining vaccine stability throughout the supply chain continues to be a challenge, especially in remote and rural areas where infrastructure and transportation are limited.

#### Opportunities

Despite these challenges, there are multiple opportunities to enhance the veterinary vaccine sector in Bangladesh.

#### Self-reliance and import substitution

The demand for veterinary vaccines continues to rise as livestock and poultry production grows. By boosting domestic production capacity, Bangladesh could cut dependency on imported vaccines, save foreign exchange, and ensure quick vaccine availability during disease outbreaks.

#### Development of advanced vaccine technologies

Modern vaccine platforms like recombinant DNA technology, cell culture–based vaccines, and thermostable formulations present significant opportunities for innovation. Thermostable vaccines for diseases such as PPR and ND could greatly enhance vaccination coverage in remote areas where cold-chain infrastructure is limited.

#### Public–private partnerships

Collaboration among LRI, universities, private pharmaceutical companies, and international organizations could speed up technology transfer, encourage innovation, and increase large-scale vaccine production. Such partnerships might also support contract manufacturing and joint venture initiatives.

#### Regional and global market opportunities

If GMP and international quality standards are fully followed, Bangladesh could potentially export veterinary vaccines to neighboring countries and other developing regions facing similar disease challenges.

#### Integration with the One Health framework

Expanding veterinary vaccine production will not only enhance livestock health but also lower the risk of zoonotic disease transmission to humans. Strengthening vaccination programs, therefore, supports global One Health goals by safeguarding animal, human, and environmental health simultaneously.

### Threats

Multiple external risks could impede the growth of domestic veterinary vaccine manufacturing.

- Heavy dependence on imported vaccines exposes the country to risks from international supply disruptions, price fluctuations, and export bans.
- Antigenic mismatches between imported vaccines and locally circulating pathogen strains can decrease vaccine effectiveness.
- Competition from well-known international vaccine brands might decrease farmer confidence in locally produced vaccines.
- Limited adoption of vaccines at the farmer level due to affordability concerns, cold-chain limitations, and lack of awareness might decrease market demand and discourage local manufacturers.

## RESEARCH AND DEVELOPMENT OF VETERINARY VACCINES IN BANGLADESH

Research and development activities for veterinary vaccines in Bangladesh are mainly managed by public institutions, especially the LRI under the DLS. The institute produces many veterinary vaccines used across the country and conducts research to improve vaccine strains, test potency, and ensure product quality. Each year, LRI produces millions of vaccine doses for national immunization programs and performs standard quality control tests like sterility, toxicity, and potency assessments before releasing vaccines.

Recently, Bangladesh has increased its research and development efforts for several key livestock vaccines, including those for PPR, FMD, LSD, ND, anthrax, and fowl cholera. These efforts are supported by collaborations among government research institutes, universities, and private sector partners.

### Landscape and institutions

LRI and BLRI serve as the main national centers for veterinary vaccine production and applied research. These organizations produce vaccines and work with universities and research laboratories to characterize pathogens, identify strains, and develop vaccines. International groups such as the World Bank and the Centers for Disease Control and Prevention help improve disease surveillance systems, vaccination logistics, and laboratory infrastructure. Meanwhile, private distributors and multinational pharmaceutical companies supply imported veterinary vaccines and biological products.

### Funding mechanisms for research and development

The landscape of research and development for veterinary vaccines in Bangladesh is primarily fueled by public-sector funding. Financial backing mainly comes from government budget allocations under the Annual Development Program, which is mainly carried out by institutions like BLRI and the DLS. However, dedicated competitive funding programs aimed specifically at veterinary vaccine innovation are still scarce.

### Translation of research into vaccine products

Academic and institutional research has produced numerous scientific publications on disease epidemiology, vaccine effectiveness, and strain analysis. However, turning these research results into licensed and commercially available vaccines remains limited. Most vaccine manufacturing still depends on incremental improvements, imported master seed strains, or technology transfer. Collaboration between universities and the pharmaceutical industry mainly occurs through project-based partnerships that involve laboratory validation, field testing, and workforce development.

### Priority diseases and research highlights

LRI produces several vaccines on a national scale to support mass vaccination programs and disease control strategies. Among these, trivalent FMD vaccines targeting serotypes O, A, and Asia-1 have been developed and tested with promising results. The Sterne anthrax vaccine produced by LRI has demonstrated protective immunity for up to one year in field trials. Research is also ongoing to evaluate the thermostable ND vaccine (I2 strain), which could help overcome cold-chain limitations in backyard poultry systems. Additional vaccine development efforts are focused on diseases such as fowl cholera, duck plague, and LSD.

### Methods and quality assurance

Current research at LRI focuses on improving antigen selection and matching for poultry vaccines through genomic surveillance and thermal stability analysis. Modernization efforts also include implementing improved laboratory and vaccine delivery systems. Quality assurance procedures adhere to standard protocols for sterility, toxicity, and potency testing. During national vaccination campaigns, vaccines produced by LRI are frequently used alongside imported vaccines to ensure an adequate supply and broader disease coverage.

## CONCLUSION

This review offers a detailed assessment of the veterinary vaccine landscape in Bangladesh by combining information on livestock disease burden, vaccine manufacturing capacity, import dependence, regulatory systems, and vaccination coverage. The analysis shows that although domestic vaccine production has grown in recent years through efforts by the LRI and various private pharmaceutical companies, the current output still falls short of meeting national demand. Locally produced vaccines now fulfill about 23%–27% of the need for ruminant vaccines, while domestic poultry vaccines make up only roughly 6%–13% of total demand. As a result, Bangladesh remains heavily dependent on imported vaccines to support its national vaccination programs.

The review further emphasizes that Bangladesh’s veterinary vaccine system is hindered by a series of layered gaps rather than a single major obstacle. The main issue is limited production capacity and technological resources, followed by regulatory and quality assurance hurdles, and ultimately operational challenges involving human resources, farmer awareness, and vaccine access. By connecting disease epidemiology, vaccine production capacity, import reliance, and field-level vaccination success, this review offers an integrated analytical framework largely missing from existing research.

The results of this review have significant implications for livestock health management, national food security, and economic stability in Bangladesh. Increasing domestic vaccine production could decrease dependence on imports, ensure better vaccine availability during outbreaks, and save foreign exchange. Improved vaccination coverage would also boost livestock productivity, minimize economic losses caused by disease, and provide greater access to animal-source protein for the growing population.

Furthermore, enhancing veterinary vaccination systems supports broader public health goals by reducing the spread of zoonotic diseases and lowering the need for antimicrobial use in livestock production. Therefore, veterinary vaccines should be seen not just as commercial pharmaceuticals but as strategic public health tools that assist national livestock development, One Health initiatives, and efforts to reduce antimicrobial resistance.

This review offers a comprehensive overview of the veterinary vaccine sector in Bangladesh by examining multiple aspects of the system, including livestock production trends, infectious disease burden, vaccination practices, domestic vaccine manufacturing capacity, import reliance, and regulatory frameworks. A major strength of the analysis is the creation of a priority-based conceptual framework that pinpoints key bottlenecks across the vaccine value chain, ranging from production technology and regulatory governance to human resource capacity and farmer level adoption. By integrating epidemiological data with production and policy insights, the review provides a holistic view of the challenges and opportunities facing the veterinary vaccine sector.

Despite its comprehensive scope, this review has several limitations. First, detailed national data on vaccine production, import volumes, and vaccination coverage are limited, and much of the available information comes from government reports, institutional publications, or sector-specific studies rather than comprehensive national databases. Second, the methods used to estimate national vaccine demand are not consistently documented in the literature, which may affect the accuracy of demand–supply comparisons. Third, there is limited publicly available information regarding vaccine pricing, economic expenditures on imports, and independent quality benchmarking of domestically produced vaccines. These data gaps emphasize the need for improved national surveillance, transparent reporting systems, and more systematic research on vaccine market dynamics.

Future progress in Bangladesh’s veterinary vaccine sector will rely on coordinated efforts across manufacturing, research, regulation, and field implementation. Modernizing vaccine production infrastructure, especially through adopting cell culture–based systems, recombinant vaccine platforms, and thermostable formulations, could greatly improve domestic capacity and technological competitiveness.

Strengthening regulatory systems is just as vital. Harmonizing GMP, establishing independent quality benchmarking mechanisms, and expanding pharmacovigilance systems would boost confidence in locally produced vaccines and encourage wider adoption.

Additionally, increased investment in human resource development and research integration is essential. Enhancing connections among disease surveillance systems, strain characterization studies, and vaccine formulation research would facilitate the creation of vaccines better tailored to locally circulating pathogens. Expanding collaboration among government agencies, universities, and private manufacturers through structured public–private partnerships could speed up technology transfer and product commercialization.

Finally, improvements at the farmer level remain crucial. Tackling issues related to affordability, cold-chain access, vaccine awareness, and farmer trust will be vital to ensuring that increased vaccine production leads to effective vaccination coverage in the field.

In summary, the veterinary vaccine sector in Bangladesh is at a critical stage of growth. While notable progress has been made in domestic vaccine production and vaccination efforts, significant gaps still exist between national demand and local manufacturing capacity. Closing these gaps requires coordinated investment in production technology, regulatory systems, research and development, and field-level vaccine distribution.

With strategic policy support, strengthened institutional collaboration, and ongoing investment in modern vaccine technologies, Bangladesh can gradually shift from a mostly import-dependent system to a more self-sufficient veterinary vaccine ecosystem. This progress would not only improve livestock health and productivity but also support national food security, economic stability, and the broader goals of the One Health framework.

## DATA AVAILABILITY

All data generated or examined throughout this review were obtained from the LRI, DLS, various private sectors, and public domain studies.

## AUTHORS’ CONTRIBUTIONS

MZH: Conceptualized and designed the review. TB and MBR: Literature review. MZH: Analyzed the data. MZH and SS: Drafted the manuscript. MZH and MMK: Edited and finalized the manuscript. All authors have read and approved the final version of the manuscript.
